# Recycling and 3D-Printing Biodegradable Membranes for Gas Separation—toward a Membrane Circular Economy

**DOI:** 10.1021/acsaenm.4c00060

**Published:** 2024-05-22

**Authors:** Sharifah H. Alkandari, Matthew Ching, Jasmine C. Lightfoot, Nael Berri, Hannah S. Leese, Bernardo Castro-Dominguez

**Affiliations:** † Department of Chemical Engineering, University of Bath, Bath BA2 7AY, U.K.; ‡ Centre for Digital Manufacturing and Design (dMaDe), University of Bath, Bath BA2 7AY, U.K.; § Centre for Bioengineering and Biomedical Technologies, University of Bath, Bath BA2 7AY, U.K.

**Keywords:** polymer membranes, gas separation, 3D printing, polymer recycling, cradle-to-cradle manufacturing, solvent-free fabrication, biopolymer

## Abstract

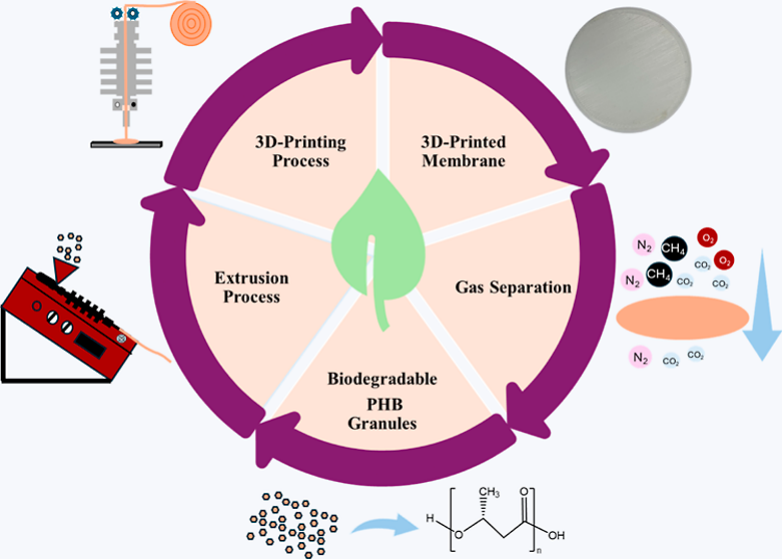

Polymer membranes employed in gas separation play a pivotal role in advancing environmental sustainability, energy production, and gas purification technologies. Despite their significance, the current design and manufacturing of these membranes lack cradle-to-cradle approaches, contributing to plastic waste pollution. This study explores emerging solutions, including the use of biodegradable biopolymers such as polyhydroxybutyrate (PHB) and membrane recycling, with a focus on the specific impact of mechanical recycling on the performance of biodegradable gas separation membranes. This research represents the first systematic exploration of recycling biodegradable membranes for gas separation. Demonstrating that PHB membranes can be recycled and remanufactured without solvents using hot-melt extrusion and 3D printing, the research highlights PHB’s promising performance in developing more sustainable CO_2_ separations, despite an increase in gas permeability with successive recycling steps due to reduced polymer molecular weight. The study emphasizes the excellent thermal, chemical, and mechanical stability of PHB membranes, albeit with a marginal reduction in gas selectivity upon recycling. However, limitations in PHB’s molecular weight affecting extrudability and processability restrict the recycling to three cycles. Anticipating that this study will serve as a foundational exploration, we foresee more sophisticated recycling studies for gas separation membranes, paving the way for a circular economy in future membrane technologies.

## 1 Introduction

Polymer membranes provide a cost-effective and environmentally friendly means to selectively separate gas mixtures by exploiting variations in gas solubilities, and/or diffusivities.^1−3^ In contrast to conventional separation methods like absorption, adsorption, and cryogenic distillation, polymer membranes offer advantages such as lower energy consumption, diminished environmental impact, and more compact equipment design.^2,3^ These distinctive features make polymer membranes indispensable for sustainable processes, encompassing carbon capture, biogas processing, and air and hydrogen purification.^4−7^

Historically, membrane development and research have prioritized functional efficiency, often neglecting environmental sustainability. Furthermore, given the typical replacement cycle of industrial membrane modules every three to five years,^8^ polymer membranes have contributed to substantial plastic waste, with nonbiodegradable materials frequently ending up in landfills or natural ecosystems.^9^ Consequently, there is a compelling need to design membranes with a cradle-to-cradle perspective, a sustainable design approach focusing on a product full lifecycle from production to reuse, ensuring that all materials can be repurposed or returned safely to the environment and considering their features throughout their life cycle.

Innovative approaches to establish a circular economy for polymer membranes involve the utilization of biodegradable polymers sourced from biological origins.^10^ Notably, cellulose acetate has undergone extensive exploration and application in gas membranes due to its distinctive attributes such as ease of processing, versatility, and eco-friendliness.^11−17^ Similarly, chitosan (CS) membranes have demonstrated effectiveness in both water treatment and gas separation.^18−21^ The molecular structure of CS, abundant in amino and hydroxyl groups, has proven to enhance CO_2_ absorption, especially in water-swollen states or when incorporated into mixed matrix membranes.^22−25^ Another illustration is provided by polylactic acid membranes, exhibiting effectiveness in biogas separation with a CO_2_ permeability of 70 Barrer and a selectivity of approximately 285 for CO_2_/CH_4_ and 26 for H_2_/CO_2_.^26,27^

In addition to the utilization of biodegradable polymers, recent research has concentrated on the recycling of end-of-life (EoL) polymeric membranes, particularly those employed in water treatment. According to Tian et al.,^28^ the recycling strategy should be tailored to the membrane condition. Severely damaged membranes undergo dissolution in organic solvents, followed by refabrication. For example, Patel et al. extracted polyvinylidene fluoride (PVDF) polymer using dimethylformamide (DMF).^29^ Wang et al.^30^ utilized *N*-methyl-2-pyrrolidone and DMF to dissolve EoL hollow fiber and flat sheet PVDF microfiltration/ultrafiltration (MF/UF) membranes. Subsequently, these membranes were reprocessed through the phase inversion method to produce recycled PVDF MF or UF membranes, demonstrating their potential as substrates for nanofiltration/reverse osmosis (NF/RO) membrane production.

For less damaged membranes, recycling occurs through regeneration, upcycling, or downcycling. Tian et al. employed PolarClean, an eco-friendly solvent, to recycle membranes used in municipal wastewater treatment.^31,32^ Some authors have embraced upcycling technology, incorporating interfacial polymerization (IP)—a polycondensation reaction—to manufacture polyamide NF/RO membranes on EoL MF/UF membranes. Recycled NF membranes have also been produced from EoL MF membranes using various techniques, including three-dimensional (3D) printing.^33−36^

The objective of this research is to design biodegradable gas separation membranes and evaluate their recycling potential using solventless mechanical methods. In this investigation, poly(3-hydroxybutyrate) (PHB) is chosen as the membrane precursor due to its status as a bioderived and biodegradable plastic.^37,38^ Moreover, it is the most common type of polyhydroxyalkanoate (PHA) variations,^39^ thus serving as a model compound for others. PHB stands out as a compelling alternative to petroleum-based polymers across a range of applications. Additionally, PHB’s compatibility with the extrusion 3D printing process allows for precise control over the membrane’s structure, which is crucial for achieving the desired gas separation performance. This selection is further justified by PHB biosynthesis from bacterial sources and its current investigation at a pilot scale for production as a byproduct of activated sludge in wastewater treatment plants,^40^ underscoring its bioderived nature and highlighting its potential integration into existing infrastructural systems for sustainable applications. Furthermore, only limited studies have explored PHB for gas separation. Follain et al.^41^ utilized homopolymer PHB and copolymer poly(3-hydroxybutyrate-*co*-3-hydroxyvalerate) or PHBV, achieving selectivity ranges of 1.5 to 6.7 for CO_2_/O_2_ and 4.8 to 19 for CO_2_/N_2_, respectively. In a separate study, Siracusa et al.^42^ observed selectivity values ranging from 1.7 to 2.5 for CO_2_/O_2_ and 2.2 to 3.4 for CO_2_/N_2_ in PHB films. Nevertheless, PHB warrants further exploration due to its environmental characteristics.

This research employs mechanical recycling through hot-melt extrusion and manufacturing via 3D printing to eliminate the use of solvents. Membrane fabrication and recycling often rely on solvents, posing risks to sustainability, operational safety, environmental impact, and human health.^43^ Additionally, 3D printing, a layer-by-layer membrane fabrication technique, offers benefits such as design flexibility, rapid prototyping, cost-effectiveness for small batches, and reduced waste. Using an advanced form of 3D printing, the membrane’s structural attributes can be precisely controlled, including thickness, surface area, and overall geometry.^44^ This technique expands the boundaries of traditional membrane fabrication, increasing the surface area by adjusting the geometry and controlling the membrane thickness.

This study marks the first systematic exploration of recycling biodegradable gas separation membranes and introduces the development of 3D-printed filaments using pristine PHB. Furthermore, it investigates the recycling potential of the polymer and its impact on the physicochemical properties. This research represents a significant step toward the development of circular membranes with improved environmental, economic, and social characteristics.

## 2 Methodology

### 2.1 Materials

**Figure 1:**
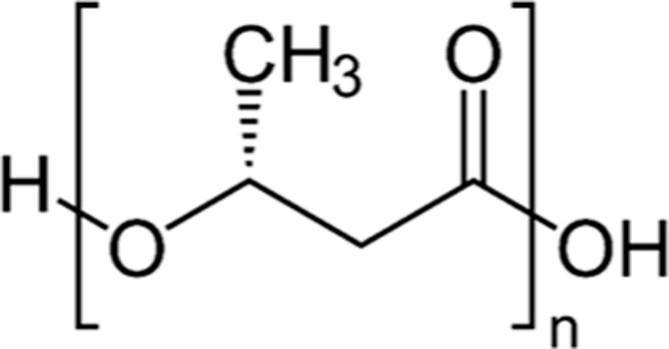
Chemical structure of PHB.

PHB used in this investigation had a molecular weight of 550 kg/mol and a granule size of 5 mm. It was purchased from Goodfellow Cambridge Ltd.—UK. The chemical structure of the PHB polymer is presented in Figure 1. To assess the barrier properties of the membranes, we used oxygen (O_2_), nitrogen (N_2_), methane (CH_4_), and carbon dioxide (CO_2_) from Sigma-Aldrich with purities of ≥99.5%.

### 2.2 Membrane Preparation

The membrane fabrication process was initiated with the drying of the as-received PHB components in a vacuum oven at 60 °C for 2 h to remove any residual moisture. Subsequently, PHB polymer granules were fed into a Noztek Touch single-screw extruder, running at 10 rpm and 165 °C, to create pure PHB filaments. It is important to note that all filaments were generated without additives. These filaments were fed to an Ender 3 Pro 3D printer equipped with a 0.4 mm nozzle. The printer generated membranes with a thickness of 0.3 and 4.7 mm in diameter. The printing process was specified to maintain nozzle and bed temperatures of 220 and 50 °C, respectively, and print at a speed of 40 mm/s. Membranes were then thoroughly characterized to understand their physicochemical properties and gas permeation properties. Finally, the membranes were cut into smaller pieces and passed through the extruder to generate new filaments. Note that membranes were not grinded as this process had a significant effect on the degradation of the polymer as described in Section 3.2.

**Figure 2:**
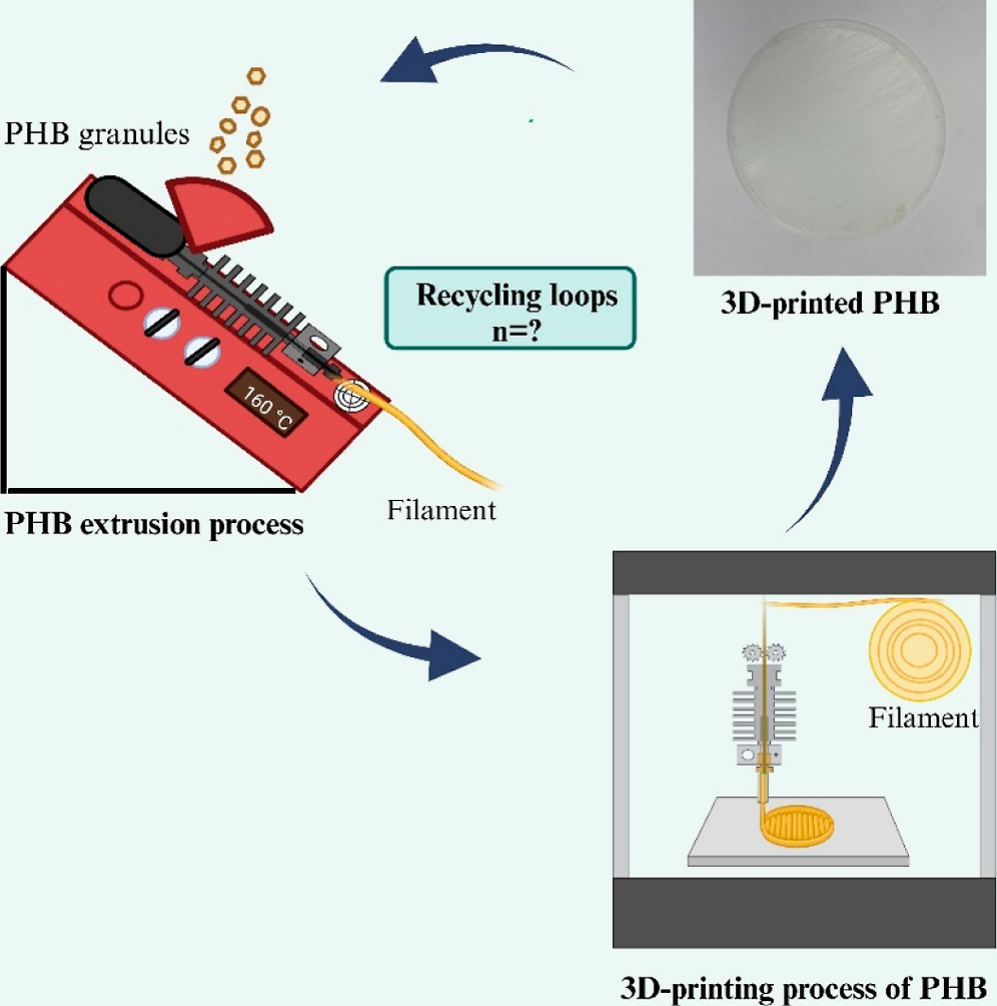
Schematic procedure of the recycling and 3D-printing cycle of PHB membranes, highlighting the closed loop from PHB granules to the printed membrane.

Figure 2 illustrates the iterative “polymer extrusion–membrane printing–membrane characterization” process employed to evaluate the recyclability of biodegradable membranes. Notably, consistent filament development in each recycling loop necessitated modifications to the extruder parameters, as detailed in Section 3.

### 2.3 Membrane Characterization

#### 2.3.1 Structural Characterization

The supramolecular structure of the membrane was analyzed by powder X-ray diffraction (PXRD). The PXRD scanning was performed on a STOE STADI P double setup operated at 40 kV and 40 mA using pure CuKα1 radiation with wavelength (λ) = 1.54059 Å. The scans ranged from 5 to 50° 2θ, with a step-size of 0.02° and a scan speed of 0.2 s per step. Likewise, the morphological characteristics of the fabricated membrane surface and cross sections were analyzed using a variable pressure scanning electron microscopy (SEM, SU3900, Hitachi, Japan).

#### 2.3.2 Chemical Characterization

Fourier transform infrared spectroscopy (FTIR, Spectrum 100 PerkinElmer USA), configured with a total reflectance cell ranging from 4000 to 650 cm^–1^ was utilized for the chemical structure analysis. The FTIR analysis was aimed at ascertaining the functional group features of the respective membranes. As a standard procedure, prior to the analysis of the actual samples, a baseline scan was conducted in transmission mode with a spectral resolution of 4 cm^–1^, recording spectra across the entire reflectance cell range.

#### 2.3.3 Thermal Characterization

The thermal characteristics (stability and degradation) of the membranes were assessed by both thermogravimetric analysis (TGA) and a differential scanning calorimeter (DSC). As a precautionary measure, prior to the thermal analysis by both TGA and DSC, the respective membrane samples were vacuum-dried overnight at 80 °C. The TGA was conducted using a thermogravimetric analyzer (TGA, TA Instruments Q-500). Approximately 20 mg of the respective membrane was fed into an alumina crucible and heated at the rate of 10 °C min^–1^, under an argon flow rate of 60 mL/min, from a temperature of 20 to 600 °C. Then, the thermal degradation, described in terms of weight loss as a function of temperature, was determined at the first derivative peak of the TGA curve, using the Setsoft 2000 thermal analysis software. The DSC analysis was done utilizing a DSC (TA Instruments Q2000), equipped with an intercooler refrigeration system. The respective membrane samples were subjected to a cycle of heating–cooling–heating at temperature ranging from −50 to 200 °C at a heating rate of 10 °C min^–1^ and nitrogen flow rate of 20 mL/min. Baseline curvature was minimized in this analysis by using an empty holder of aluminum as a reference in the alternative sample holder of the DSC. The degree of crystallinity (*X*_c_) was determined using the following eq 1.
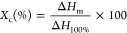
1where Δ*H*_m_ is the melting enthalpy per unit of weight of the PHB sample and Δ*H*_100%_ denotes the enthalpy per unit weight of the 100% crystalline PHB, which is assumed to be 146 J/g.

#### 2.3.4 Gel Permeation Chromatography

The Agilent gel permeation chromatography (GPC) and size exclusion chromatography (SEC) analysis was performed using an Agilent Infinity II MDS instrument configured with a differential refractive index, viscometry, dual-angle light scatters (LS), and variable wavelength UV detectors. The configuration of the equipment consisted of 2 × PLgel Mixed D columns (300 × 7.5 mm) and a PLgel 5 μm guard column. CHCl_3_ was used as the eluent, and ethanol was added as a flow rate marker. Prior to the actual sample analysis, the system was calibrated between 535 and 1,591,000 g mol^–1^ using poly(methyl methacrylate), and polystyrene standards (Agilent EasiVials). The respective samples to be analyzed were solubilized overnight, then filtered through a 0.22 μm pore size nylon membrane prior to injection. The samples were analyzed at a flow rate and temperature of 1 mL min^–1^ and 30 °C, respectively. Critical parameters of the PHB polymer, including dispersity (PD), molecular weight mass average (*M*_w_), and number-average (*M*_n_) were determined before and after membrane fabrication by using Agilent GPC/SEC software.

#### 2.3.5 Mechanical Property Analysis

The mechanical properties of the respective PHB membranes were analyzed according to the ASTMD 882 standard method using an INSTRON (3369, England) instrument at room temperature. The following fabricated membranes PHB-1, PHB-2, and PHB-3 were investigated for tensile stress, tensile strain at break, and tensile modulus. The tensile strength is equal to the ratio of the maximum load to the cross-sectional area. The elongation of the polymer films was evaluated as the percentage of elongation. This was done by taking the ratio of the change in length and the original length of the films, expressed as a percentage. A total of three samples from each cycle were subjected to testing. In order to minimize the inaccuracy resulting from material overlap during the process of 3D printing, tension was intentionally applied in alignment with the printing direction (Figure S5).

#### 2.3.6 Gas Sorption Analysis

The BET surface area and pore size distribution of the PHB, PHB-1, PHB-2, and PHB-3 were analyzed to assess the formation of any micropores. This was done through nitrogen adsorption and desorption measurements at 77 K, using a Micromeritics 3Flex 3500 volumetric gas sorption analyzer. The specific surface area was calculated by employing the Brunauer–Emmett–Teller (BET) method. This involved regression analysis of relative pressure data ranging from 0 to 1.0, in accordance with the guidelines provided by the manufacturer.

#### 2.3.7 Gas Permeation Test

Single gas (CO_2_, O_2_, CH_4_, and N_2_) permeation tests of the membranes were measured by means of a constant volume/variable pressure time-lag apparatus. The gases were fed at both fixed pressure and variable pressure values at 25 °C, and permeability was evaluated from the steady-state rate of pressure increase at a fixed downstream volume. A detailed description of the procedure and experimental setup is presented.^45^

## 3 Results and Discussion

### 3.1 PHB Filament Manufacturing for 3D Printing

This study centered on producing PHB membranes via 3D printing, involving the creation of PHB filaments from both fresh granules and recycled PHB 3D-printed membranes. Table 1 outlines the temperature and motor speed utilized during each extrusion cycle. The initial membrane, PHB-1, was crafted from fresh PHB granules at 165 °C and 10 rpm, yielding high-quality, consistent filaments. After PHB-1 characterization, the membranes were re-extruded, using the same extruder process parameters, to produce similar quality filaments. These filaments were used to produce PHB-2, and the recycling continued with the temperature reduced to 155 °C for optimal 3D printing in PHB-3. However, due to changes in the mechanical properties of PHB on re-extrusion, the polymer melt was not sufficiently viscous to be processed following a third extrusion at 145 °C.

**Table 1:** Parameters of Extruded PHB Polymer Filaments

PHB polymer membrane	extrusion temperature (°C)	extruder motor speed (rpm)
PHB granule	165	10
first cycle of the printed membrane, PHB-1	165	10
second cycle of the printed membrane, PHB-2	155	10
third cycle of the printed membrane, PHB-3	145	10 (unsuccessful)

**Figure 3:**
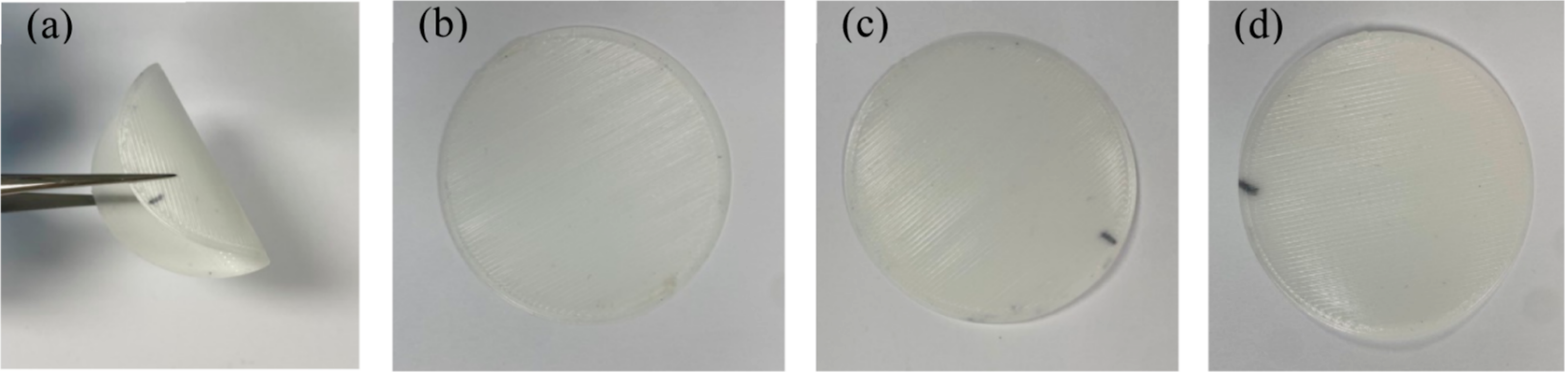
3D-printed PHB polymeric membranes: (a) illustration of PHB-1 flexibility; (b) PHB-1; (c) PHB-2; (d) PHB-3.

Each recycling step exhibited a decreased viscosity, necessitating lower extrusion temperatures. Despite this, all fabricated membranes, including those from recycled PHB, displayed ease of handling, high flexibility, and flawless surfaces, as depicted in Figure 3. Notably, no distinguishable differences were observed in the surfaces or coloration between membranes produced from original PHB granules and the recycled PHB polymer.

### 3.2 GPC: Molecular Weight Changes During Recycling

GPC was used to characterize PHB and determine the molecular weight distributions at different recycling loops. Table 2 shows the values for *M*_n_, *M*_w_, and the polydispersity index (PDI) (calculated as *M*_w_/*M*_n_) of the different recycled membranes. *M*_n_ represents the statistical average molecular weight of all polymer chains in a sample, which can be estimated through polymerization mechanisms or ascertained by techniques that assess the quantity of molecules in a specific weight sample, like colligative methods, including end-group assay. In contrast to *M*_n_, *M*_w_ incorporates the molecular weight of each chain when calculating the average molecular weight, meaning that heavier chains have a more significant impact on *M*_w_. *M*_w_ is measured using methods focusing on molecular size instead of just the number of molecules, such as light scattering techniques.^46^

**Table 2:** Summary of Polymer Properties, *M*_n_, *M*_w_, and the PDI of Neat PHB Granules and Ground PHB and PHB Membranes

membrane samples	*M*_n_ (g/mol)	*M*_w_ (g/mol)	PDI
PHB granule		550,000	
PHB grinded	60,975	142,712	2.3
PHB-1	86,121	224,699	2.6
PHB-2	68,358	200,559	2.9
PHB-3	63,657	186,303	2.9

**Figure 4:**
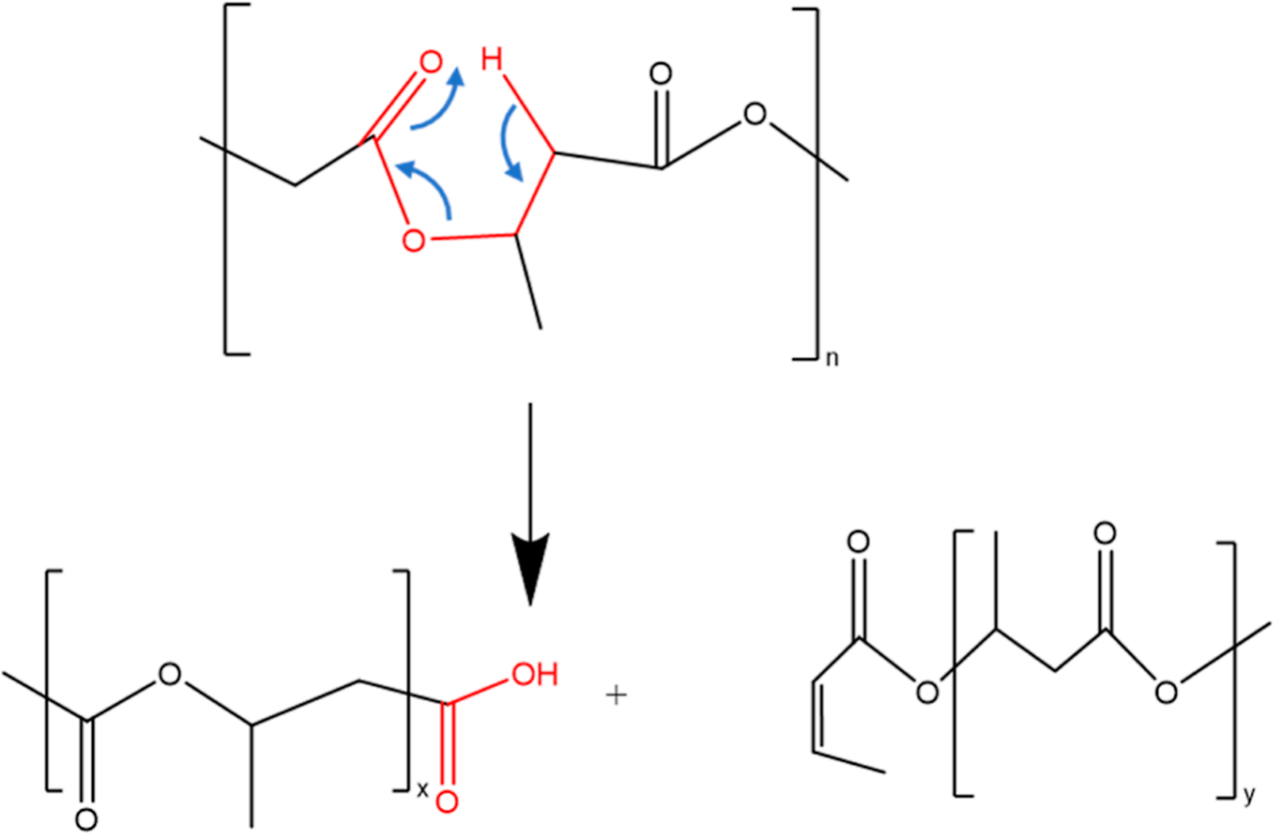
Schematic of the thermal degradation of PHB by random chain scission.

The results show that the raw PHB granules have the highest *M*_w_. Upon thermal recycling, the polymer chains were shortened, reducing the *M*_w_. This observation is consistent with those reported across the literature.^47,48^ The observed reduction in *M*_w_ and *M*_n_ is attributed to polymer chain scission, as depicted in Figure 4, which shows a shortening of the polymer chains. Chain scission results in fewer entanglements and a decrease in the viscosity across successive recycling cycles. Concurrently, the PDI value increased after each cycle, indicating that chain scission led to a more varied distribution of chain lengths within the polymer.

Although all experiments to produce the membranes in this work were subject to melt extrusion; as a control experiment, raw PHB granules were subject to a grinding process to assess the effect of mechanical action on the reduction of the molecular weight. The results showed a significant reduction in *M*_w_ due to chain scission caused by the grinding process. In contrast, as shown in Table 2, recycled membranes revealed that the 3D printing process resulted in no significant reduction in *M*_w_, even after multiple recycling cycles, when compared to grinding. Although PHB undergoes a dual-extrusion process during its application in 3D printing; initially, PHB is extruded to create the filament, which is subsequently used in the 3D printing process and then extruded again during printing. This repeated exposure to high temperatures may adversely affect the material’s thermal stability. This implies that the mechanical force from the grinding process impacted the polymer more than did the thermal effect. This assertion is supported by the conclusions reported in previously published studies.^48−50^

### 3.3 Structural Characterization

The XRD spectra of the neat PHB granule, and the respective cycles of recycled PHB are presented in Figure S1. The diffractograms show similar crystalline characteristics depicting orthorhombic crystalline planes,^51,52^ with three characteristic crystalline peaks at 2θ 13.5, 16.9, and 25.5° assigned to the (020), (110), and (130) planes of the orthorhombic unit cell. This observation is consistent with the findings acknowledged by the Joint Committee on Powder Diffraction Standards (JCPDS).^53^ In addition, several other weaker reflections were observed in the spectra. For instance, the peak at a 2θ of 21.02°, which is assigned to reflection of the (021) plane shows that the respective 3D-printed PHB membrane spectra have some amount of orthorhombic β-form crystal with zigzag conformation. The manifestation of the β-form crystal could contribute to the appearance of a shoulder prior to the melting peak on the DSC, which is similar to the observation reported in the referenced study.^52^ The XRD images show that the crystallinity of the 3D-printed membrane was not significantly affected by the recycling process. This finding is supported by the appearance of characteristic crystalline peaks for PHB, PHB-1, PHB-2, and PHB-3, all displaying the same 2θ values.

**Figure 5:**
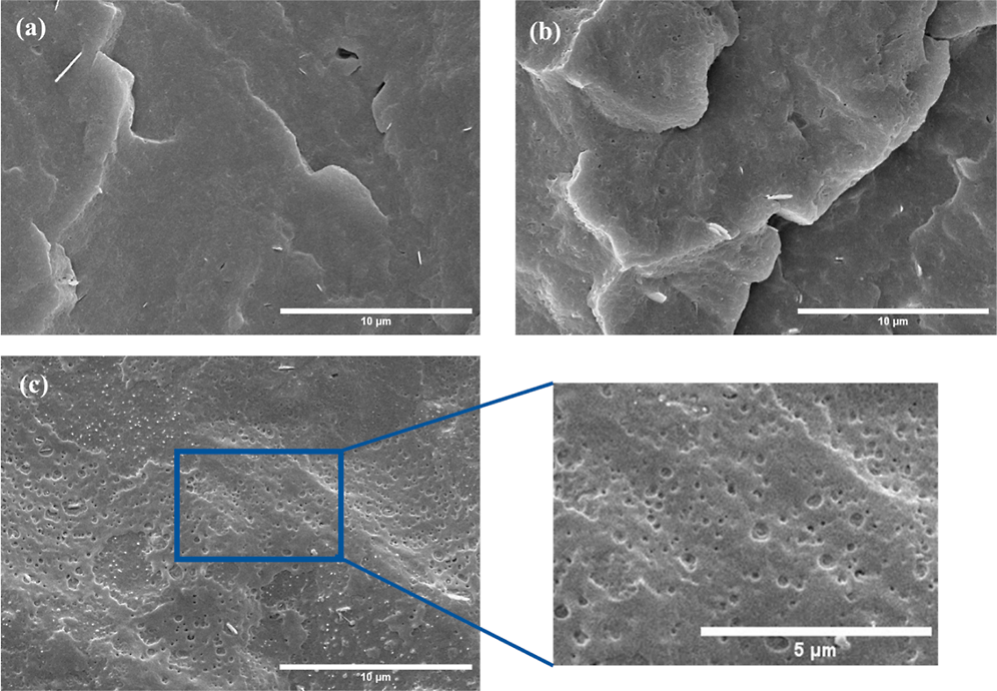
SEM images for (a) PHB-1, (b) PHB-2, and (c) PHB-3 cross section.

The morphological structure of each PHB membrane was examined via SEM to assess any potential changes in the membranes. Figures S2 and 5 display the surface and cross-section SEM images, respectively, of the PHB-1, PHB-2, and PHB-3 membranes. The PHB-1 membrane, with a higher molecular weight, displayed a defect-free, smooth surface with clear layering lines characteristic of 3D printing (Figure S2a_1_). Layering lines on the membrane surface lead to an increase in surface area compared to conventional flat membranes.^54^ 3D printing can therefore enable membrane design optimization for superior gas separation performance. PHB-1 membrane’s cross-section (Figure 5a) showed a dense structure, suggesting tightly packed polymer chains typical of high molecular weight polymers. This density is attributed to chain entanglements limiting mobility and resulting in a compact formation.^55−57^Figure S2b_1_ shows a defect-free surface for the PHB-2 membrane. However, voids or pore structures were observed in the cross-section (Figure 5b), indicating a less dense polymeric structure compared to the PHB-1 membrane. This could be due to a lower molecular weight, reducing chain entanglements and consequently increasing the number of chain ends. The GPC analysis in Table 2 revealed that membrane PHB-2 has lower *M*_n_ and *M*_w_ compared to PHB-1, which implies more chain ends that are more mobile and less tightly packed than the middle parts of the chain. As a result, areas near the chain ends tend to have more free volume and allow for a more open structure.^56,58,59^ This trend was also observed on PHB-3 membranes, which displayed the lowest molecular weight values (Figure 5c).

To confirm the formation of pores, BET surface areas of PHB-1, PHB-2, and PHB-3 were determined through low-pressure nitrogen gas adsorption at −195 °C. The resulting BET surface areas were found to be 1.05 m^2^/g for PHB-1, 1.18 m^2^/g for PHB-2, and 1.28 m^2^/g for PHB-3. With each recycling cycle, the observed gradual rise in surface area may indicate the formation of additional voids (Supporting Information, S1).

### 3.4 Chemical Characterization

FTIR analysis was performed on the membranes to assess any potential changes in the chemical structure of the polymer. Figure S3 displays the FTIR spectra of the PHB granule and different membranes. The scanning range for FT-IR was 500–4000 cm^–1^, revealing several key features. A prominent absorption band at 1720 cm^–1^ was observed, representing the ester carbonyl group, aligned with the C=O stretching bond within the molecular chain. Between 1163 and 1210 cm^–1^, a series of absorption bands indicated ester group C–O bond stretching. The bending vibrations seen at 2969 and 2932 cm^–1^ were indicative of the presence of the methyl group, and the peak at 1378 cm^–1^ was associated with the methyl group symmetric bending. The band at 1454 cm^–1^ suggested the asymmetric bending of the −CH_2_ and −CH_3_ groups. Moreover, a moderately intense band at 3435 cm^–1^ was identified as belonging to the hydroxyl group, which agrees with a typical FTIR spectrum of PHB reported in literature^51,60^ The findings imply that recycling the polymer did not alter its chemical properties nor introduce any new chemical components.^61^

### 3.5 Thermal Characterization

**Figure 6:**
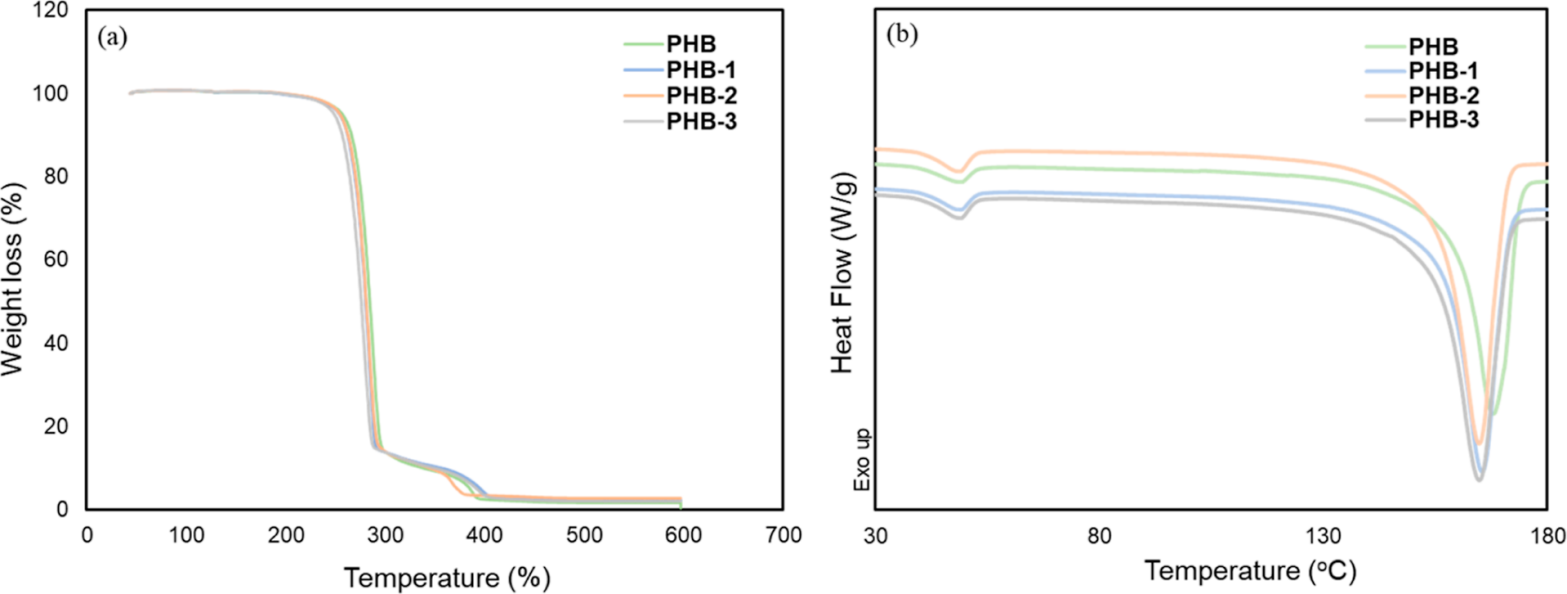
Thermal analysis of membranes: (a) TGA; (b) DSC curves for the 2nd heating cycle.

The evaluation of the thermal properties of the fabricated PHB membranes was conducted by using TGA and DSC, as illustrated in Figure 6. The TGA shows no major differences between samples with an initial thermal degradation phase between 235 and 293 °C, resulting in an 85% reduction in weight. Further degradation was observed between 293 and 400 °C, resulting in an additional 11% weight loss. PHB exhibited a significant thermal degradation at 400 °C, corresponding to a weight loss of 96%. Also, a minor shift is observed at the beginning of the curve after each cycle, as depicted in Figure 6a. Notably, the degradation curves of the respective PHB membrane display comparable patterns as those previously published in literature and their characteristic temperatures remain consistent.^41^

Figure 6b shows the DSC curves for the second heating cycle run performed during heating up to 180 °C for the 3D-printed PHB granule, PHB-1, PHB-2, and PHB-3, respectively. The thermal behavior of these membranes followed a similar trend. The main melting peaks were observed at *T*_m_ = 161 °C for the PHB granule and at *T*_m_ ranging from 156 to 158 °C for the respective recycled membranes (PHB-1, PHB-2, and PHB-3). It can be noted that after each recycling process, there was a gradual reduction of the melting temperature. This decrease in *T*_m_ can be attributed to the high temperatures and shear stresses encountered during 3D printing and the extrusion process, which lead to chain scission or the breaking of molecular chains in the polymer (Figure 4). This phenomenon is evident from the data in GPC result Table 2. Indeed, polymers with a lower molecular weight typically exhibit lower melting temperatures as they have fewer molecular entanglements and reduced intermolecular forces, thereby facilitating easier movement of the chains relative to each other and enabling a smoother transition to a liquid state.^62,63^Table 3 shows the values of the thermal properties (*T*_cc_—cold crystallization; *T*_m_—melting point for the first and second heating cycle; Δ*H*_m_—Enthalpy for the first and second heating cycle, and *X*_c_ %—degree of crystallinity). It was observed that the *T*_cc_ and Δ*H*_m2_ is stably maintained for all the membranes and occurred at approximately *T*_cc_ = 109 °C and Δ*H*_m2_ = 70 J/g. The *T*_cc_ is primarily influenced by the ability of polymer chains to arrange into a crystalline structure. These findings support the observation previously stated that the recycling of the PHB polymer did not alter the regularity of the polymer chains and, as such, did not impact the crystallization rate.

**Table 3:** Summarized Crystallinity of Membranes

membrane	*T*_m1_ (°C)	*T*_m2_ (°C)	Δ*H*_m1_ (J/g)	ΔH_m2_ (J/g)	*T*_cc_ (°C)	*X*_c_ %
PHB	163	161.5	71.2	76.1	109.3	52.1
PHB-1	158	157.7	70.7	75.6	109.1	51.7
PHB-2	157	156.5	68.6	75.5	108.9	51.7
PHB-3	157	156.1	70.9	75.2	109.7	51.5

### 3.6 Membrane Mechanical Properties

Table 4 presents a comprehensive summary of the mechanical properties of recycled PHB membranes, displaying Young’s modulus, tensile strength at break, and elongation at break. The initial data reveal that PHB-1 possesses a Young modulus of 3052 MPa, which exhibits a gradual decrease in subsequent membranes PHB-2 and PHB-3. Young’s modulus is a critical measure, reflecting the material resistance to deformation under stress, essentially quantifying the ratio of stress to strain. The observed reduction in Young’s modulus can be attributed to the polymer chain scission occurring with each recycling cycle. Longer polymer chains, which are more effective in stress transfer, give way to shorter chains, resulting in decreased stiffness and a lower Young modulus. Additionally, the repeated heating process during recycling may lead to the degradation of key structural elements of the polymer.

**Table 4:** Mechanical Properties of Fabricated PHB Membranes

membranes	Young’s modulus (MPa)	tensile strength at break (MPa)	elongation at break (%)
PHB-1	3052	18.8	3.1
PHB-2	2963	17.7	2.7
PHB-3	2954	16.3	2.5

**Figure 7:**
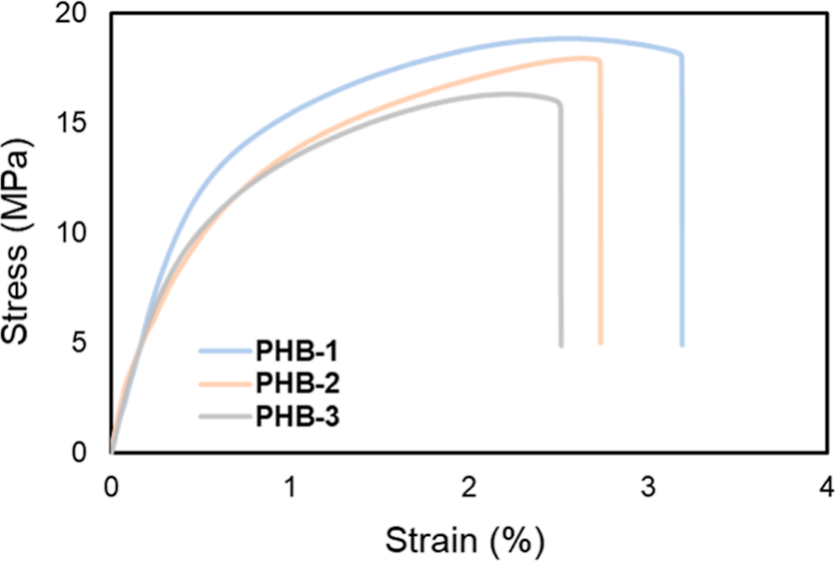
Tensile stress vs strain curve of PHB membranes.

Moreover, the observed data indicate a minor reduction in both tensile strength and elongation at break. This reduction corresponds to a decrease in softness and flexibility when subjected to mechanical stress, as depicted in Figure 7. This decline in mechanical properties can be attributed to repeated recycling, which is expected to shorten polymer chains, resulting in a less compact structure with fewer entanglements and a reduced molecular weight, as explained by refs (47,48, and 64) These changes, primarily resulting from polymer chain scission and structural degradation during recycling, highlight the challenges in maintaining the mechanical integrity of recycled polymers. This understanding is crucial for optimizing recycling processes and enhancing the performance of recycled materials in practical applications.

### 3.7 Gas Permeation Tests

**Figure 8:**
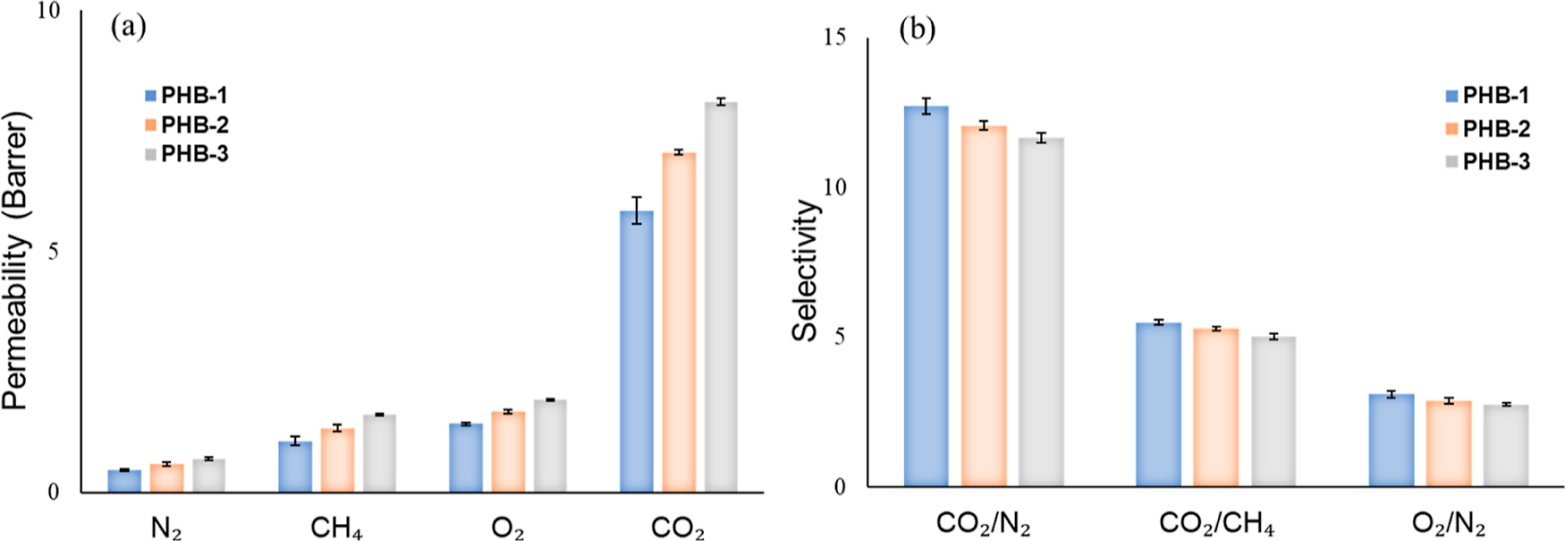
Pure gas permeability and ideal gas selectivity.

Figure 8a displays the outcomes of single gas permeation tests (N_2_, CH_4_, O_2_, and CO_2_) conducted at 25 °C with a constant 3 bar feed pressure. This test evaluated the impact of recycling cycles on the separation performance of 3D-printed membranes created using the PHB polymer recycled at different cycles. PHB-1 displayed a CO_2_ permeability of 6 Barrer (0.02 GPU) and a selectivity of 13, 6, and 3 for CO_2_/N_2_, CO_2_/CH_4_, and O_2_/N_2_, respectively. PHB-2 revealed a modest enhancement in CO_2_ permeability to 7 Barrer (0.023 GPU). However, this was coupled with a discernible decrease in selectivity, yielding values of 12, 5.2, and 2.8 for CO_2_/N_2_, CO_2_/CH_4_, and O_2_/N_2_, respectively. Lastly, PHB-3 demonstrated the highest CO_2_ permeability among the tested membranes at 8.2 Barrer (0.027 GPU). Nonetheless, this was concomitant with a further reduction in selectivity.

The physicochemical properties of a polymer, such as mechanical, thermal, viscosity, permeability, and others, are dictated by two elements: (a) chemical composition and (b) supramolecular structure.^65,66^ By demonstrating that the polymer did not undergo any chemical change (Figure S3), we can conclude that any change in permeability is due to changes in the supramolecular structure. Supramolecular structure is affected by polymer chain lengths, polymer crystallinity, and solvent/additive effects.^67−69^ As crystallinity remained the same (Figure S1) and no additional additives or solvents were used in the process, the data suggest that the cause for permeability changes is due to changes in molecular weight.

Gas transport through dense polymeric membranes is governed by the solution-diffusion mechanism, wherein a gas molecule initially adsorbs at the interface between the feed and the membrane, then diffuses through the membrane, and finally desorbs on the permeate side. Consequently, permeance is influenced by two key factors: solubility and diffusivity, both of which are a function of the membrane materials properties.^70,71^ Gas solubility is influenced by factors such as the condensability of the gas, indicated by its critical temperature (*T*_c_), and the interaction between the gas and polymer chains. Similarly, gas diffusivity is impacted by several elements, including the size of the gas molecule as measured by its kinetic diameter (*D*_K_), the membrane fractional free volume, the interaction between gas and polymer chains, and the flexibility of the polymer chains.^72^ The results indicated that the gas permeability of the membrane progressively increased as the number of PHB polymer recycling cycles increase, following the sequence PHB-3 > PHB-2 > PHB-1. As the number of recycling cycles increases, the polymer chains are shortened, as indicated by a reduction in the molecular weight (Table 2); this, in turn, increases the free volume at the molecular level, allowing for quicker diffusion of small gas molecules.^73,74^ The permeability differences for N_2_, CH_4_, O_2_, and CO_2_ between the PHB-1 and PHB-3 membranes were 46, 47, 30, and 37%, respectively. Additionally, the results showed that the membranes exhibited size-selective behavior, with permeability decreasing as the kinetic diameter of the gas increased (CO_2_ > O_2_ > CH_4_ > N_2_). This observation aligns with other research findings and comparable with polymers of a similar type and structure, like other PHAs, as reported in various sources.^41,42,49^ Notably, CO_2_ permeability remained significantly high across all recycled membranes, largely due to the predominant positive effect of solubility,^49^ smaller kinetic diameter, and higher critical temperature (*T*_c_ = 31.05 °C and *D*_K_ = 0.33 nm). Owing to these factors, CO_2_ gave a better solubility and diffusivity than CH_4_ (*T*_c_ = −82.45 °C and *D*_K_ = 0.38 nm), O_2_ (*T*_c_ = −118.55 °C and *D*_K_ = 0.346 nm), and N_2_ (*T*_c_ = −147.05 °C and *D*_K_ = 0.364 nm), thereby leading to promising selectivity values for CO_2_/N_2_ and CO_2_/CH_4_.^75^ Given that the gas permeability values improved with the increase in PHB recycling cycles for the three recycled PHB membranes, it is concluded that membrane gas permeability is not adversely affected by polymer recycling.

In contrast, the selectivity of the membrane pair gases (CO_2_/N_2_, CO_2_/CH_4_, and O_2_/N_2_) gradually decreased with an increase in the number of PHB polymer recycling cycles, following the order PHB-3 < PHB-2 < PHB-1, as shown in Figure 8b. The decrease in CO_2_/N_2_, CO_2_/CH_4_, and O_2_/N_2_ selectivity between the PHB-1 and PHB-3 membranes was 9, 9, and 12%, respectively. As previously mentioned, the recycling cycles lead to a rearrangement of polymer chains, creating more free volume, and some defects and voids begin to emerge, reducing the gas selectivity of the membrane. This is due to morphological changes, as demonstrated in the cross-sectional SEM images of PHB-2 and PHB-3 (Figure 5). Nevertheless, it can be inferred from the observed increase in gas permeability and the slight reduction in ideal selectivity that polymer recycling can effectively be utilized in creating polymer membranes without significantly compromising their general properties. Moreover, as illustrated in the Robeson upper bound plot (Figure S7), the membrane’s performance aligns with the permeability and selectivity demonstrated by numerous biodegradable polymers.^41,49,76^ Given these characteristics, the PHB membranes explored in this study emerge as eco-friendly and solvent-free alternatives in comparison to their counterparts. This performance positions them as promising candidates for sustainable CO_2_ separations.

### 3.8 Physical Aging and Stability Tests

In this study, PHB-3 membranes were exposed to a single feed of CO_2_ gas at 3 bar pressure for 360 h continuously to evaluate the membrane stability. The results revealed a slight reduction in CO_2_ permeability after 360 h, amounting to only 3%. This suggests that the sample was stable throughout the test. This finding indicates that recycled PHB membranes are likely to retain their effectiveness without significant aging under similar continuous CO_2_ feed conditions. The resistance of the membrane against physical aging may be attributed to the constant occupation of free volume within the polymer matrix by CO_2_ molecules, which restricted the movement of the polymer chains, as suggested in the literature.^77^

**Figure 9:**
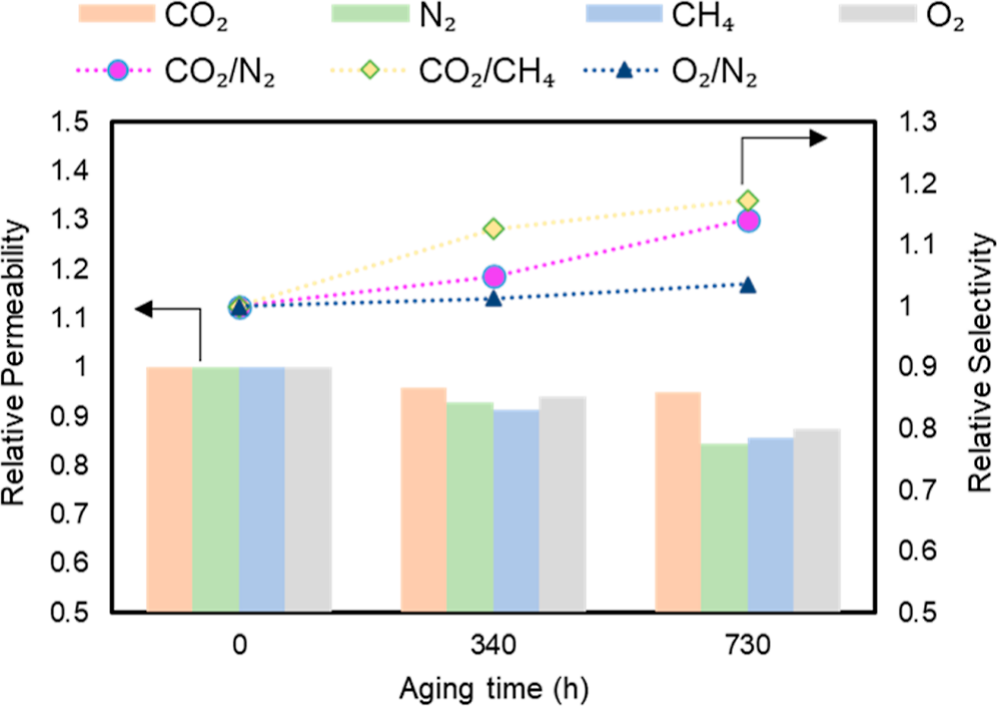
Relative permeability and relative ideal selectivity over time of the PHB-3 membrane.

Another membrane stability test was conducted for the PHB-3 membrane, and the gas permeability of all species (CO_2_, O_2_, CH_4_, and N_2_) was tested periodically for 730 h. In between tests, the membrane was stored in dry air. Figure 9 shows that the PHB-3 membrane displayed a slight decrease in the relative permeability for all gases. Notably, the CO_2_ permeability diminished by 4% within the first 340 h, with a further 1% decrease observed between 340 and 730 h. Conversely, N_2_, O_2_, and CH_4_ showed a more pronounced reduction in permeability compared to CO_2_, therefore, enhancing the membrane selectivity for CO_2_/CH_4_, CO_2_/N_2_, and O_2_/N_2_.

The reduction in permeance is attributed to an increase in polymer crystallinity over time as PHB undergoes significant cold-crystallization and structural rearrangement due to its glass transition temperature being below room temperature (*T*_g_ ∼ 2 °C).^78,79^ When PHB is stored at or above its *T*_g_, the polymer chains acquire enough mobility to gradually form crystallites. However, over time, at temperatures above *T*_g_, PHB can undergo annealing, allowing the chains to reorient and evenly distribute across the material. Even so, we recommend long-term stability tests to thoroughly understand the impact of physical aging and degradation over time on the gas permeability through PHB.

## 4 Conclusions

This study focuses on creating 3D-printed gas separation membranes using PHB, an environmentally friendly and biodegradable polymer, while investigating their recycling potential. The recycling method involves extrusion to recycle PHB membranes, followed by their reconstruction via 3D printing with each recycling cycle undergoing thorough characterization at every phase.

Our findings indicate that successive recycling rounds of PHB membranes led to decreased polymer chain length, directly impacting their thermal, mechanical, and gas permeation traits. Recycled membranes displayed heightened gas permeability but suffered a decline in gas selectivity. Nonetheless, these membranes exhibited stability during extended testing (over 300 h). This study underscores several key discoveries pertinent to the engineering of circular and sustainable gas separation membranes:

(1) PHB, a biodegradable and sustainable polymer, can create 3D-printed membranes without the need for additives.
(2) PHB membranes demonstrate promising performance for more sustainable CO_2_ separations.
(3) 3D printing proves to be a reliable method for manufacturing consistent PHB membranes.
(4) The mechanical recycling of PHB membranes to produce new ones results in reduced polymer chain length with subsequent effects on physicochemical properties.
(5) The gas permeation and selectivity of recycled PHB membranes suffer slight changes, deemed minor for gas separation applications.

## Supporting Information Available

Characterization of the 3D-printed PHB membranes including XRD patterns, FT-IR spectra, SEM, DSC, and nitrogen adsorption–desorption isotherms for 3D-printed PHB granules and recycled PHB membranes (PDF)
