# Supplementary material for: Recycling and 3D-Printing Biodegradable Membranes for Gas Separation—toward a Membrane Circular Economy
Source: ACS Appl Eng Mater. 2024 May 22;2(6):1515–25. doi: 10.1021/acsaenm.4c00060 (PMC11217943; doi:10.1021/acsaenm.4c00060)
Supplement: Supplementary file 1 — em4c00060_si_001.pdf [file em4c00060_si_001.pdf]

Supporting Information

Recycling and 3D-Printing Biodegradable Membranes for Gas Separation - Towards a Membrane Circular Economy

Sharifah H. Alkandari[a], Matthew Ching[a], Jasmine C. Lightfoot[a], Nael Berri[a,c], Hannah S. Leese[a,c], and Bernardo Castro-Dominguez[a,b,*]

[a]*Department of Chemical Engineering, University of Bath, Bath BA2 7AY, United Kingdom.*
[b]*Centre for Digital Manufacturing and Design (dMaDe), University of Bath, Bath BA2 7AY, United Kingdom.*
[c]*Centre for Bioengineering and Biomedical Technologies, University of Bath, Bath BA2 7AY, United Kingdom.*

[*] **Corresponding author:** Email: bcd28@bath.ac.uk; Office Number: +441225384946

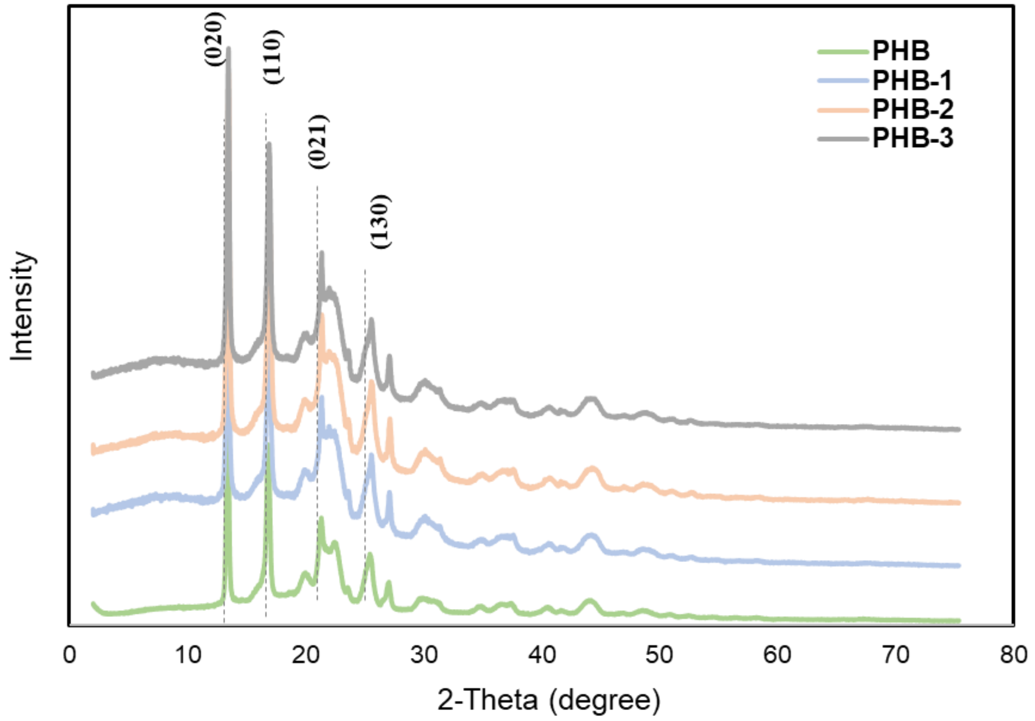

Figure S1: XRD pattern for 3D printed PHB granule and recycled PHB membranes.

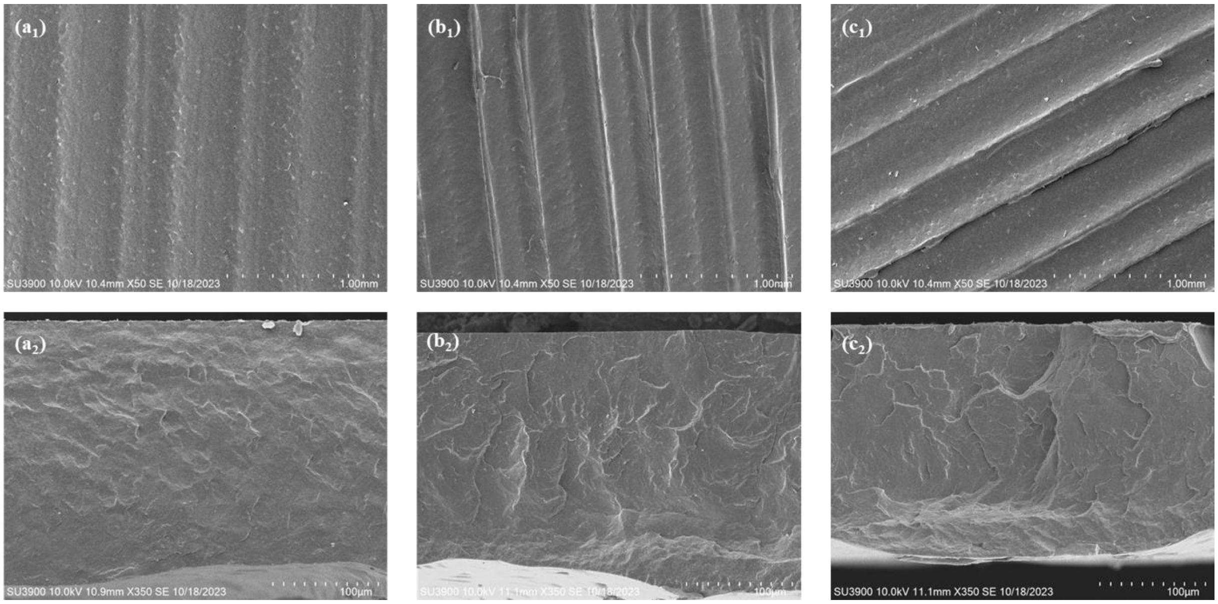

Figure S2: SEM images for: (a_1) PHB-1 surface, (a_2) PHB-1 cross-section (b_1) PHB-2 surface, (b_2) PHB-2 cross section, (c_1) PHB-3 surface, (c_2) PHB-3 cross section.

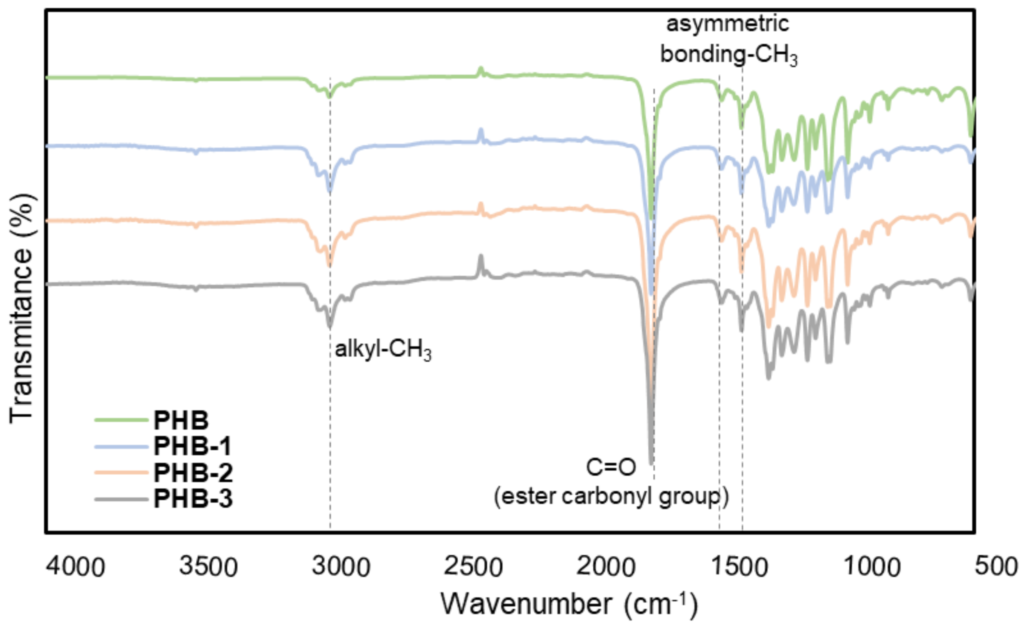

Figure S3: FTIR analysis for PHB granule and 3D printed membranes.

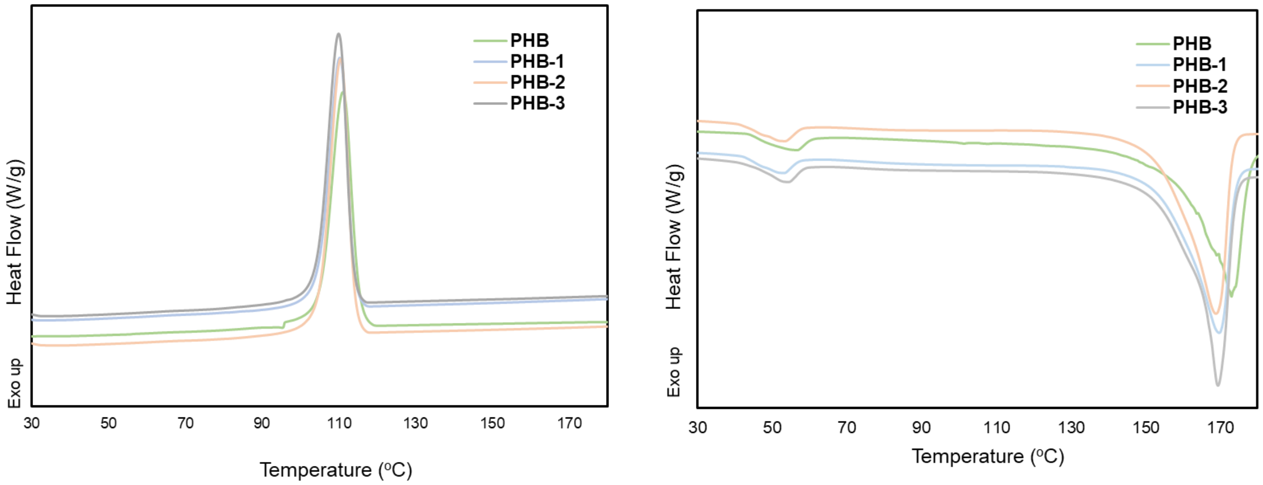

Figure S4: Thermal analysis of membranes: (a) DSC curves for cooling cycle and (b) DSC curves for 1st heating cycle.

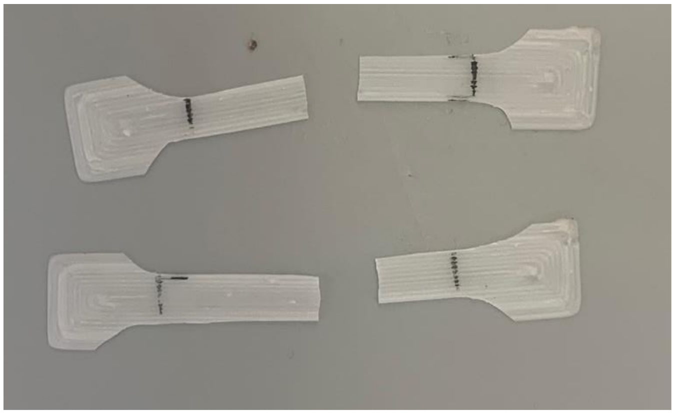

Figure S5: tested dog-bone samples.

S1. Gas Sorption Analysis

The impact of the recycling process on the morphology and structure was further investigated by conducting nitrogen adsorption-desorption isotherms on PHB-1, PHB-2, and PHB-3, as depicted in Figure S6. The isotherm profiles for all three membranes are remarkably consistent, suggesting comparable structures and surface areas. The similarity of the curves implies that the membranes are either uniformly dense or possess similar distributions of voids, as marked discrepancies in porosity would manifest in more varied isotherm shapes. The measured BET surface areas for PHB-1 PHB-2, and PHB-3 were 1.05m^2/g, 1.18 m^2/g, and 1.28m^2/g, respectively. The increase in surface area with each recycling iteration could suggest the introduction of more voids, which in turn would present more surface for gas adsorption, thereby increasing the measured surface area. Nonetheless, the BET method may not yield the most definitive characterization for highly dense membranes, as the relatively low surface area values could be within the instrument margin of error.

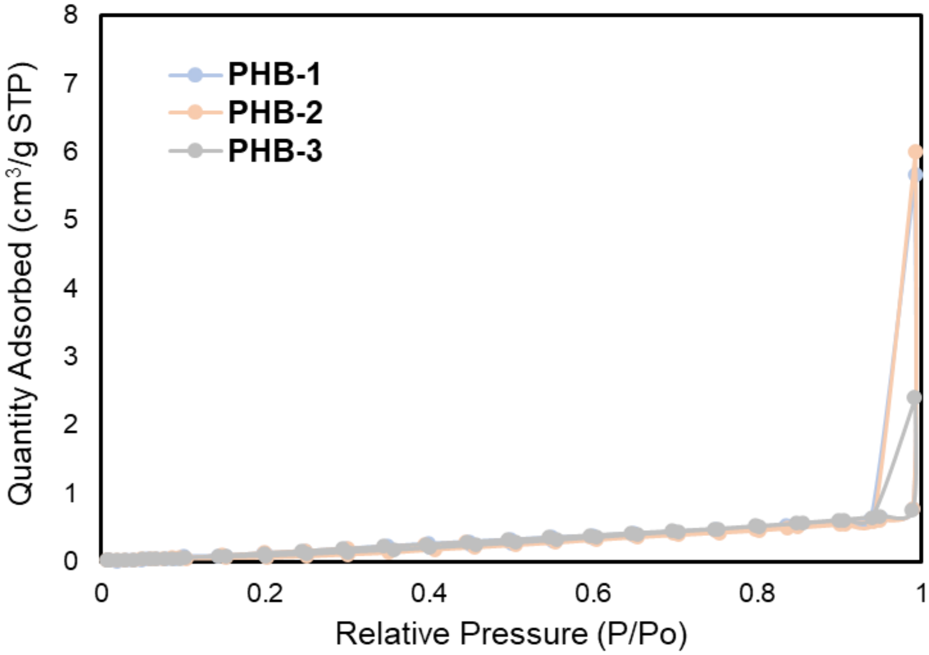

Figure S6: Nitrogen adsorption−desorption isotherms.

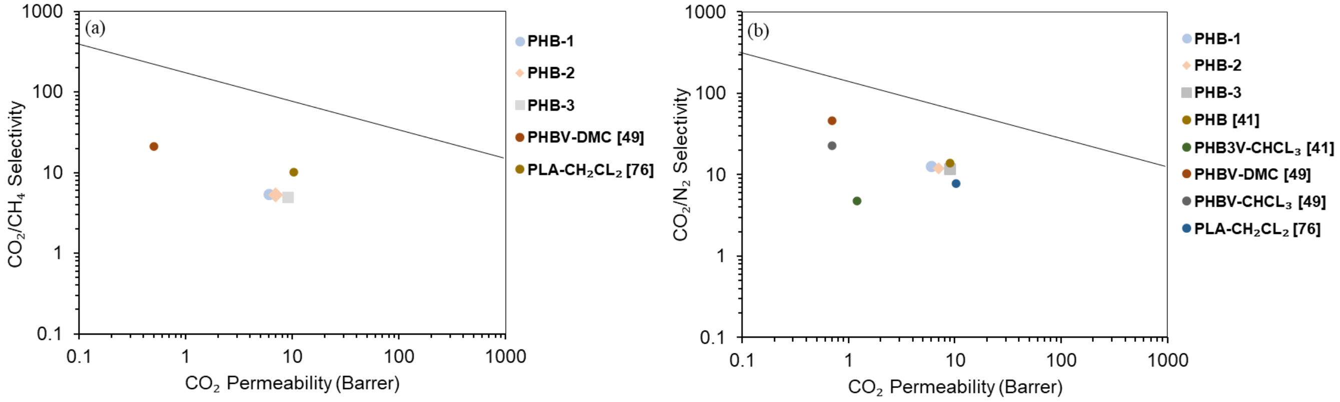

Figure S7: Robeson Upper bound for (a) CO_2/CH_4 and (b) CO_2/N_2 for of PHB, PHB-1 and PHB-3 with other bio-based polymeric membranes studies.
